# Optimal Offloading Decision Strategies and Their Influence Analysis of Mobile Edge Computing

**DOI:** 10.3390/s19143231

**Published:** 2019-07-23

**Authors:** Jiuyun Xu, Zhuangyuan Hao, Xiaoting Sun

**Affiliations:** College of Computer Science and Technology, China University of Petroleum (East China), Qingdao 266580, China

**Keywords:** MEC, computation offloading, optimal offloading decision

## Abstract

Mobile edge computing (MEC) has become more popular both in academia and industry. Currently, with the help of edge servers and cloud servers, it is one of the substantial technologies to overcome the latency between cloud server and wireless device, computation capability and storage shortage of wireless devices. In mobile edge computing, wireless devices take responsibility with input data. At the same time, edge servers and cloud servers take charge of computation and storage. However, until now, how to balance the power consumption of edge devices and time delay has not been well addressed in mobile edge computing. In this paper, we focus on strategies of the task offloading decision and the influence analysis of offloading decisions on different environments. Firstly, we propose a system model considering both energy consumption and time delay and formulate it into an optimization problem. Then, we employ two algorithms—Enumerating and Branch-and-Bound—to get the optimal or near-optimal decision for minimizing the system cost including the time delay and energy consumption. Furthermore, we compare the performance between two algorithms and draw the conclusion that the comprehensive performance of Branch-and-Bound algorithm is better than that of the other. Finally, we analyse the influence factors of optimal offloading decisions and the minimum cost in detail by changing key parameters.

## 1. Introduction

With the development of mobile communication technologies and the popularity of smart devices, a wide variety of network devices and applications emerge in an endless stream. The need for network performance, such as time delay, is getting higher and higher. At the same time, the processing power of mobile devices is also getting stronger and stronger but they still cannot handle those applications requiring great computing power. In addition, processing these applications locally faces another problem, namely the rapid consumption of battery power. Especially for those applications that need real time replay such as online games or Virtual Reality (VR) games, traditional cloud computing architecture does not work well in these fields due to relatively remote geographical distance. These issues severely impact the application’s efficiency and user experiences. In order to solve the above problems, the industry has proposed mobile edge computing (also known as Fog Computing). In 2016, European Telecommunications Standards Institute (ETSI) extended the concept of mobile edge computing (MEC) to Multi-access Edge Computing, extending Edge Computing from telecom cellular networks to other wireless Access networks (such as WiFi). In short, mobile edge computing processes workload from wireless devices locally(such as routers, eNodeB etc.) instead of sending the workload to remote cloud servers. Note that the edge computing does not aim to perfectly substitute cloud computing but to complement it. Edge computing just extends cloud computing to the network edge [1]. In belief, the ‘fog’ is a cloud but closer to the ground.

Although edge computing can provide a rapid response, the capacity of the edge server is still limited. It is difficult to process the compute-intensive task (e.g., face recognition, video encoding, etc.) for the edge server. It is impractical to accomplish those tasks by relying on the edge server alone. In contrast to the edge server, the cloud server has far more computing power than the edge server but the cloud server cannot provide a rapid response. So we can combine cloud computing with edge computing in the real production environment. Making the optimal offloading decision therefore becomes critical. Bad strategy can cause network congestion, energy waste and task timeouts. In other words, we need to decide where the task is executing. An important contribution of this paper is building a mathematical model considering both edge server and cloud server in making the optimal offloading decisions. In addition, the combination of edge and cloud can save a mount of economic cost in Internet of Things (IoT). According to the Wikibon IoT Project, Cloud + Edge Computing is 36% of the cost of Cloud-only Computing when the reduction in data volume is 95%.

**Figure 1:**
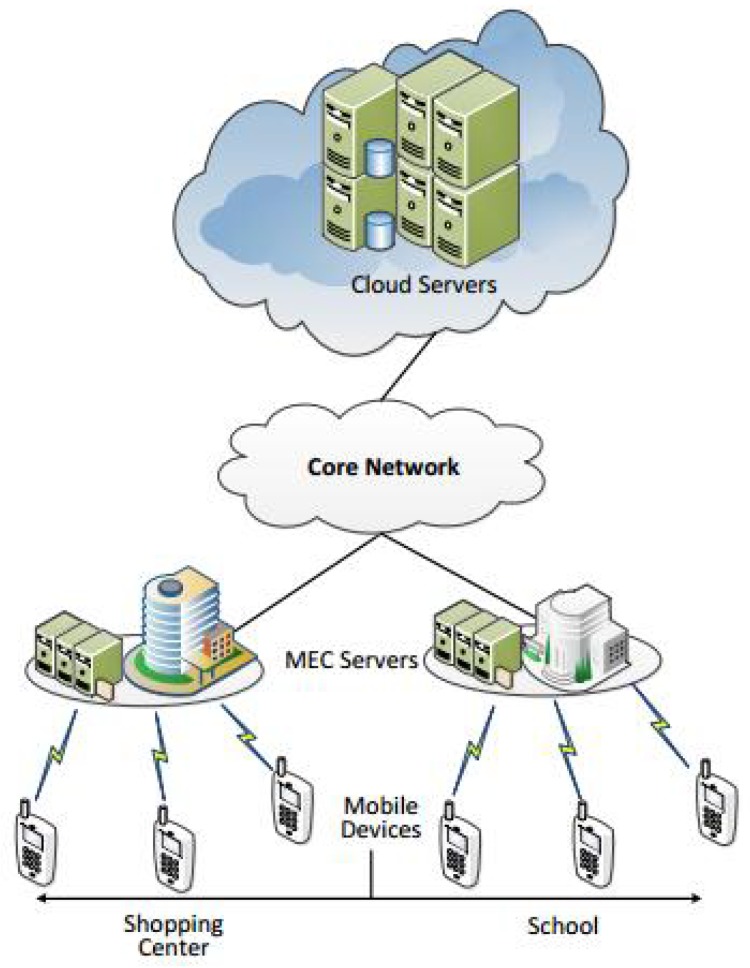
Mobile Edge Computing Architecture.

As shown in Figure 1, this architecture is composed of a lot of cellular networks and a core cloud server with unlimited computational capacity. The cellular networks have some wireless devices (WDs) that are directly connected to a base station with strong but still limited computational capacity. The WD’s computation task can be executed in three ways: executing locally, offloading it to the edge server and offloading it to the cloud server. For simplicity, we use ‘thing computing’, ‘edge computing’ and ‘cloud computing’ to represent them respectively. This architecture is called Combined Fog-Cloud (CFC) [2,3] or Fog-to-Cloud (F2C) [4]. A typical application of this architecture is Vehicular Networking (VN) [5]. In the VN the fog servers are hosted by Road Side Units (RSU). In this way, the fog servers (RSU) can provide single-hop mobile links for vehicles to achieve lower delay and delay-jitter compared to directly connecting to a remote cloud server. This will greatly improve the Quality of Service (QoS) of VN applications. In this paper, we will formulate a system model and employ two methods to obtain the optimal or near-optimal decision to minimize the system cost, including the time delay and energy consumption in one cellular network.

Although MEC is not as mature as cloud computing [6], there has been a lot of research on MEC. Deng [3] first mathematically formulates the task offloading decision problem. It decomposes the primal problem into three sub-problems of corresponding subsystems, which can be independently solved. The author compared the energy consumption and system delay between cloud computing, edge computing and cloud-edge computing. In addition, Huang [7], Li [8], Deng [9] and Kao [10] built the mathematical model and formulate it into an optimization problem to get the minimum system cost including time delay and energy consumption. Li [8] has proposed a Deep Reinforcement Learning based algorithm to tackle task offloading in MEC. Huang [7] has proposed a deep learning based algorithm for MEC and it uses multiple parallel Deep Neural Networks (DNNs) to generate offloading decisions. Both of them make use of artificial intelligence (AI) technology and achieved significant results. Xavi [4] has proposed a layered MEC model and introduced the advantages and disadvantages of this architecture and Xavi [4] focuses on the coordinated management of MEC. There is much similar research, such as in References [11,12,13,14,15,16]. All of them solve a binary computation offloading problem in nature, namely the architecture they have proposed only includes cloud server or edge server. To meet the needs of the production environment, we must combine cloud computing with edge computing according to the previous discussion. In addition, all the above authors only focus on how to make the optimal offloading decision and do not analyse the influence of model parameters variation on offloading decisions in detail. In this paper, we study the variation of optimal offloading decisions and minimize system costs based on three types of offloading decisions (executing locally, edge computing and cloud computing) under different key parameters. Although the real MEC environment is more complex than the mathematical model proposed in this paper, the simulating result still has directive significance to a certain degree. The main contributions of this paper are highlighted as follows:

- We propose a fog-cloud system model considering both energy consumption and time delay and formulate it into an optimization problem.We employ two algorithms, Enumerating and Branch-and-Bound, to get the optimal or near-optimal decision for minimizing the system cost including the time delay and energy consumption.
- We compare the performance of two algorithms and draw the conclusion that the comprehensive performance of the Branch-and-Bound algorithm is better than that of the other.
- We analyse the influence factors of optimal offloading decisions and the minimum cost in detail by changing key parameters and the analysis results can direct real production.

The rest of this paper is organized as follows. In Section 2, we present the system model including the network model, task model and computing model. In Section 3, we described the formulation of our optimization problem. In Section 4, we introduce our method in detail. In Section 5, we show the simulation results. Finally, we conclude this study in Section 6.

## 2. System Model

### 2.1. Network Model

Based on Figure 1, we only study one cell (an area served by one fog server), the set of WDs in the same cell is denoted as N={1,2,…,N} and we use *n* to represent the WD in the set N whose index is n. Based on N, we proposed an offloading action vector A and defined it as $A=\{a_{1},a_{2},\ldots ,a_{n}\}$ where $a_{n}$ represent the offloading decision, respectively—namely the real offloading decision of WD *n*. We denote $a_{n}\in \left\{0,1,2\right\}$ to represent three conditions (executing locally, edge computing and cloud computing) respectively, so we have $a_{n}=0$, $a_{n}=1$, $a_{n}=2$ to represent local execution, edge computing and cloud computing, respectively. The computation capacity of WD *n* is denoted as $f_{n}^{l}$ (the CPU frequency (Ghz)). Accordingly, the computation capacity of Edge Server and Core Cloud Server are denoted as $f^{e}$ and $f^{c}$, respectively.

We assume the bandwidth between WD *n* and the edge server is denoted as $b_{n}^{w}$. Besides, we assume the bandwidth of edge server, core network and core cloud server is large enough so the bandwidth does not become the bottleneck, which means the transport delay (the time transmitting data from network card to the transmission media) produced by the edge server, core network and core cloud server can be neglected but the propagation delay (the time of signal propagation from end to end) of the core network cannot be neglected because the geographical distance between edge server and cloud server is relatively far in general. For simplicity, we assume that the propagation delay from the fog server to the cloud server is equal to the propagation delay from the cloud server to the fog server. So we use *t* to represent the total propagation delay of the core network in a cloud computing. In the real system, we can get *t* by *ping* command or other more precise method. According to Chen [11] and Zhang [17] the download data rate is very high in general and the data size of the result is much smaller than that of input data, so the delay and energy consumption at download step are neglected in the rest of this paper.

### 2.2. Task Model

We assume that every WD has one task to process at the same time, we use $B_{n}$ to represent the size of the data to be transferred. We use $D_{n}$ to denote the task size expressed as the total number of CPU cycles required to accomplish the computation task. $D_{n}$ reflects the amount of computing resource required to finish the task. There is a liner relation between $B_{n}$ and $D_{n}$ [8]. It can be represented as

(1) $$B_{n}=\theta D_{n}$$

We assume that whether executed by UE *n* locally or on the MEC server, the size of $D_{n}$ remains the same.

We assume the task cannot be divided into partitions to be processed on different devices, which means that each WD should execute its task by local computing or offloading computing.

### 2.3. Computation Model

(1) Local Computing Model: If the *n*-th WD chooses to execute its task locally, we define $T_{n}^{l}$ as the local execution delay of WD *n* which only includes the processing delay of local CPUs. The local execution delay $T_{n}^{l}$ is

(2) $$T_{n}^{l}=\frac{D_{n}}{f_{n}^{l}}$$

Then, we define $E_{n}^{l}$ as the energy consumption when executing its task locally. The $E_{n}^{l}$ is

(3) $$E_{n}^{l}=T_{n}^{l}*P_{d}=\frac{D_{n}*P_{d}}{f_{n}^{l}}$$

where $P_{d}$ represents the power of a Wireless device when a task in a wireless device is executed locally and we assume the power of WDs is identical.

Combining the time (2) and energy (3) cost, the total cost of local computing can be given as

(4) $$Q_{n}^{l}=\alpha E_{n}^{l}+\beta T_{n}^{l}$$

where α and β represent the weights of time and energy cost of task and the weights satisfy 1≤α≤100,1≤β≤100.

(2) Edge Computing Model: If WD *n* chooses to offload a task and execute it on the edge server, we define $T_{n}^{e}$ as the time cost of the edge computing of the WD *n* when it chooses to offload a task to the edge server and it consists of two parts, $T_{n,t}^{e}$ and $T_{n,p}^{e}$. The $T_{n,t}^{e}$ is the transmission delay and it is expressed as

(5) $$T_{n,t}^{e}=\frac{B_{n}}{b_{n}^{w}}$$

And $T_{n,p}^{e}$ is the processing delay of edge server, it can be represented as

(6) $$T_{n,p}^{e}=\frac{D_{n}}{f^{e}}$$

according to (5) and (6) the $T_{n}^{e}$ is

(7) $$T_{n}^{e}=T_{n,t}^{e}+T_{n,p}^{e}$$

Similarly, the energy cost of edge computing $E_{n}^{e}$ is composed of the transmission energy consumption $E_{n,t}^{e}$ and the idle consumption of WD $E_{n,i}^{e}$ as well, and their representation as flow

(8) $$E_{n,t}^{e}=T_{n,t}^{e}*P_{t}$$

(9) $$E_{n,i}^{e}=T_{n,p}^{e}*P_{i}$$

where $P_{t}$ is the transmission power of the WD *n* and $P_{i}$ is the idle power and we assume both are constants. So the $E_{n}^{e}$ is

(10) $$E_{n}^{e}=E_{n,t}^{e}+E_{n,i}^{e}$$

Combining the time (7) and energy (10) cost, the total cost of offloading computing can be given as

(11) $$Q_{n}^{e}=\alpha E_{n}^{e}+\beta T_{n}^{e}.$$

(3) Cloud Computing Model: If WD *n* chooses to offload a task and execute it on the cloud server, we define $T_{n}^{c}$ as the time cost and $E_{n}^{c}$ as the energy cost under this policy. $T_{n}^{c}$ still includes $T_{n,t}^{c}$ and $T_{n,p}^{c}$. Note that the $T_{n,t}^{c}$ is equal to the $T_{n,t}^{e}$. In addition, $T_{n}^{c}$ includes an extra delay caused by data transmission between the edge server and core cloud server. Because the geographical distance between the edge server and core cloud server is so far that the delay cannot be neglected, generally speaking. So we define it as a constant *t* because the transport delay can be neglected. We define $T_{n,p}^{c}$ as

(12) $$T_{n,p}^{c}=\frac{D_{n}}{f^{c}}.$$

So, $T_{n}^{c}$ is

(13) $$T_{n}^{c}=T_{n,t}^{c}+T_{n,p}^{c}+t$$

the energy cost of cloud computing is very similar to this in edge computing. So, for simplicity, we directly give the formula as

(14) $$E_{n,t}^{c}=T_{n,t}^{c}*P_{t}$$

(15) $$E_{n,i}^{c}=\left(T_{n,p}^{c}+t\right)*P_{i}$$

(16) $$E_{n}^{c}=E_{n,t}^{c}+E_{n,i}^{c}$$

Combining the time (13) and energy (16) cost, the total cost of cloud computing is

(17) $$Q_{n}^{c}=\alpha E_{n}^{c}+\beta T_{n}^{c}$$

and the sum cost of all users in the MEC offloading system is expressed as

(18) $$Q_{all}=\sum_{n=1}^{N}\left(\frac{\left(1-a_{n}\right)\left(2-a_{n}\right)}{2}Q_{n}^{l}+a_{n}\left(2-a_{n}\right)Q_{n}^{e}+\frac{a_{n}\left(a_{n}-1\right)}{2}Q_{n}^{c}\right)$$

we assume there are *N* WDs in the cellular network. We interpret the Formula (18) by simulating a scene with four wireless devices, that is N=4. In this scene, device 1 chooses to offload tasks to the cloud server, that is $a_{1}=2$, device 2 chooses to offload tasks to the edge server, that is $a_{2}=1$, device 3 and device 4 choose to execute locally, that is $a_{3}=0,a_{4}=0$. We use (18) to calculate the total power consumption, that is, $Q_{all}=Q_{1}^{c}+Q_{2}^{e}+Q_{3}^{l}+Q_{4}^{l}$. All of the important notations used in this paper can be found in Table 1.

## 3. Problem Formulation

In this paper, to minimize the total cost include delay and energy cost with our system model is to minimize $Q_{all}$. The minimum cost is denoted by $V_{min}$. In this formula there are two variables we have taken into account. They are $a_{n}$—offloading mode and $b_{n}^{w}$—the bandwidth between the WD *n* and the edge server. So, we can formulate an optimization problem to minimize $Q_{all}$, which is expressed as follows:

(19) $$\begin{array}{c} V_{min}=\underset{A,B}{minimize}\;\sum_{n=1}^{N}\left(\frac{\left(1-a_{n}\right)\left(2-a_{n}\right)}{2}Q_{n}^{l}+a_{n}\left(2-a_{n}\right)Q_{n}^{e}+\frac{a_{n}\left(a_{n}-1\right)}{2}Q_{n}^{c}\right)\\ \begin{array}{ccc} s.t.\;C1: & \sum_{n=1}^{N}b_{n}^{w} & \leq \lambda \\ C2: & b_{n}^{w} & \geq 0,\forall n\in N\\ C3: & a_{n} & \in \{0,1,2\},\forall n\in N \end{array} \end{array}$$

where $A=[a_{1},a_{2},\ldots ,a_{n}]$ is the the offloading decision vector and $B=[b_{1}^{w},b_{2}^{w},\ldots ,b_{n}^{w}]$ is the bandwidth allocation. C1 represents the total bandwidth in this system are limited and the total up-link bandwidth allocated for all users cannot exceed the maximum bandwidth λ. If computation tasks are aggressively offloaded to the edge server or cloud server, a severe congestion will occur on the uplink wireless channels, which leads to a significant delay in executing computation tasks. So it is necessary to limit the bandwidth.C2 represents the bandwidth allocated to every WD is can not be negative. C3 represents the offloading decision just has three modes and only takes three values 0, 1 and 2.

The optimization problem (19) is a mixed-integer programming problem, which is difficult to solve in general. Traditional heuristics and evolutionary algorithms(e.g., Particle Swarm Optimization (PSO), Genetic Algorithm (GA), etc.)can not directly solve this problem. In this paper, we employ two methods to solve this optimization problem (19). One is an enumerating algorithm, the other is a Branch and bound algorithm. In the following Sections the detail of these two methods showing as follows.

## 4. Problem Solution

### 4.1. Enumerating Algorithm

The reason mixed-integer programming problems is difficult to solve is there are some integer variables in the objective function, nonlinear or integer programming algorithms alone cannot handle this. In other words, if we take those integer variables with determined values, namely we provide the offloading mode vector A, the origin optimization problem (19) becomes a common nonlinear programming problem only with the bandwidth allocation vector B:

(20) $$\begin{array}{c} \underset{B}{minimize}\;\sum_{n=1}^{N}\left(\frac{\left(1-a_{n}\right)\left(2-a_{n}\right)}{2}Q_{n}^{l}+a_{n}\left(2-a_{n}\right)Q_{n}^{e}+\frac{a_{n}\left(a_{n}-1\right)}{2}Q_{n}^{c}\right)\\ \begin{array}{ccc} s.t.\;C1: & \sum_{n=1}^{N}b_{n}^{w} & \leq C\\ C2: & b_{n}^{w} & \geq 0,\forall n\in N \end{array} \end{array}$$

where, towards a common nonlinear programming problem, the python library scipy can solve this kind problem reliably. So we can produce all possible offloading modes in one decision process, then use scipy to produce corresponding optimal values for every possible offloading mode and choose the minimum one as the output. The detailed algorithm is shown in Algorithm 1. We called this algorithm the Enumerating Algorithm (EA).

**Algorithm 1:** Enumerating Algorithm.

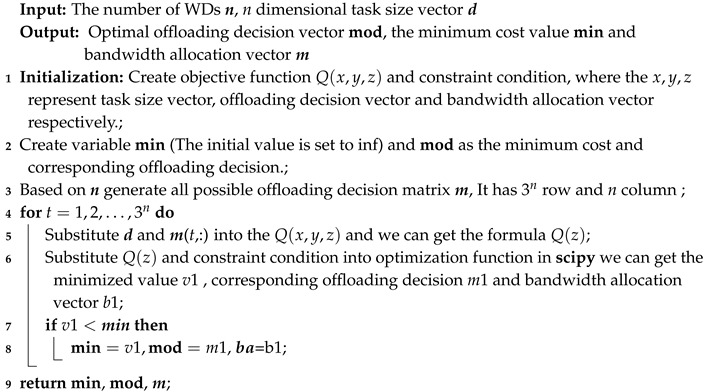



The advantage of this algorithm is obvious, that is, it can get a more reliable solution. In this paper, we consider it the global optimal value but this enumerating algorithm has a serious disadvantage—the time complexity of this algorithm is $O(3^{n})$, namely time increases exponentially with the number of tasks. It is unsuitable for a scenario with a large number of devices but the Branch and Bound algorithm can get the result relatively rapidly. The time consuming comparison between Enumerating Algorithm and Branch and Bound algorithms is shown in Section 5.

### 4.2. Branch and Bound

**Figure 2:**
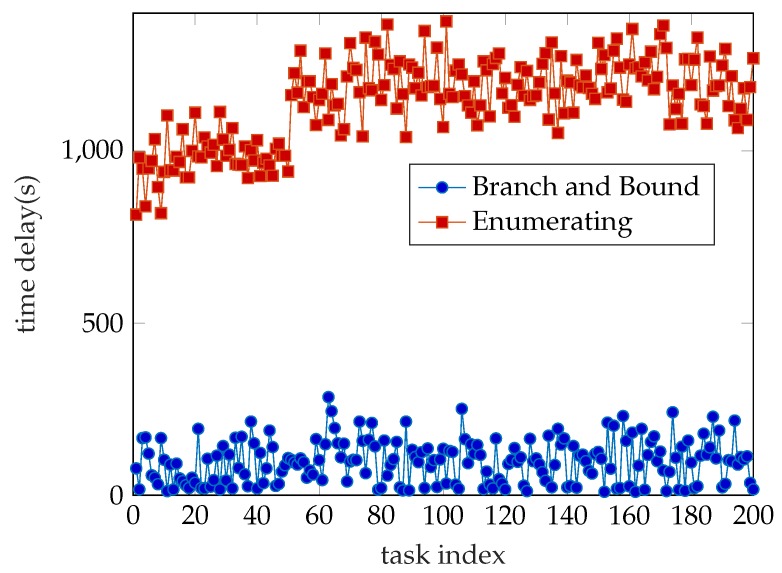
Time consumption.

Branch and bound is one of the most commonly used algorithms for integer programming problems. This method can solve not only pure integer programming but also mixed integer programming. The branch and definition is to search all feasible solution Spaces of the constrained optimization problem (whose feasible solution is finite number) properly. The total solution space is usually iteratively divided into smaller and smaller subsets called branches and a target lower bound (for the minimum problem) is calculated for the solution set in each subset, which is called bound. After each branch, if the target value of a known feasible solution set cannot reach the current limit, then the subset is discarded. Thus, many subsets are not considered, which is called pruning. This is the idea of the Branch and Bound. More detailed information about the algorithm can be found on Wikipedia. In this work, we use an improved third-party Matlab function to solve this mixed-integer programming problem (19) directly. We only need to convert (19) into matlab structure and put it into the Branch and Bound function to solve it. Then we can get the approximate optimal value and corresponding offloading decision and bandwidth allocation. The time complexity of this algorithm is between O($n^{2}$) and O($2^{n}$). Furthermore, our experimental results show that the time consumption of the Branch and Bound Algorithm is only one tenth of the Enumerating Algorithm. This experimental result is shown in Figure 2. We discuss the precision of this algorithm in Section 5.

## 5. Simulating Results and Analysis

In this section, we use the above two proposed algorithms to solve this mixed-integer programming problem (19) and get the numerical results. Then we compare the performance of the two algorithms. We also study the variation trend of the total cost and offloading decision through multiple simulations. For the setting of system model parameters, we have referred to References [7,8]. In our simulation, we set the number of WD as 9 and assume every device has one task to make a offloading decision. Then we set the $f_{n}^{l}$, $f^{e}$ and $f^{c}$ as 1000 MHz, 3000 MHz and 5000 MHz respectively. In addition, we set α=10,β=1,θ=100,t=0.25,W=150. At last, The $P_{d}$, $P_{t}$ and $P_{i}$ are set as 0.5 W, 0.3 W and 0.1 W, respectively. We vary the task size $B_{n}$ from 500 to 5000. The experimental environment is:

- CPU:Intel(R) Core(TM) i7-4790 CPU@3.60 GHz
- Memory: 8 G DDR3 1600 MHz
- OS: Windows 8.1

### 5.1. Performance Comparison

**Figure 3:**
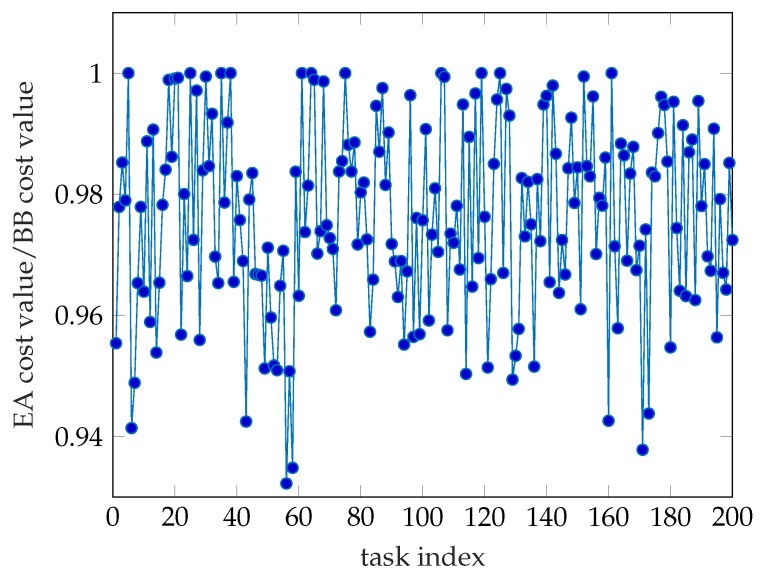
The accuracy rate of the Branch and Bound algorithm.

Time consumption is an important indicator for evaluating algorithm performance. As shown in Figure 2, we have computed 200 tasks and the result is quite clear. The time consumption of the Branch and Bound (BB) algorithm is much less than the Enumerating Algorithm’s. The average time cost of both algorithms is 96.9 s and 1142.8 s, respectively. The accuracy rate is another important indicator. In this paper, we use the cost value generated by the Enumerating Algorithm as the minimum value and consider the corresponding offloading decision as the optimal decision. So, we use the ratio of the cost value obtained by Enumerating algorithm to the cost value obtained by Branch and Bound algorithm as the accuracy rate of the Branch and Bound algorithm and is shown in Figure 3. We can see that the lower accuracy rate of the Branch and Bound algorithm is higher than 93% and the average accuracy rate acquired by statistics is 97.63%. It means we only lose the negligible accuracy rate but we get more than tenfold arithmetic speed compared to the EA. Note that the result is produced by the System model which only possesses 9 WDs, that is, there is a rapidly growing time consumption gap between BB and EA as the number of WDs on the same cellular network increases. In large-scale simulations and industrial applications, for the proposed mixed-integer programming problem, the Branch and Bound algorithm is much better than the Enumerating Algorithm.

The BB algorithm also can apply to a similar edge computing model to get the optimal offloading decision if it can be formulated into a mixed-integer programming problem, such as References [7,8,18]. A comparison between the BB and Enumerating Algorithm can be found in References [7,8,18] and we will carry on the research in the future work.

In the following experiment, all the experimental results are generated by the Branch and Bound algorithm.

### 5.2. Influence Analysis of Model Parameters

**Figure 4:**
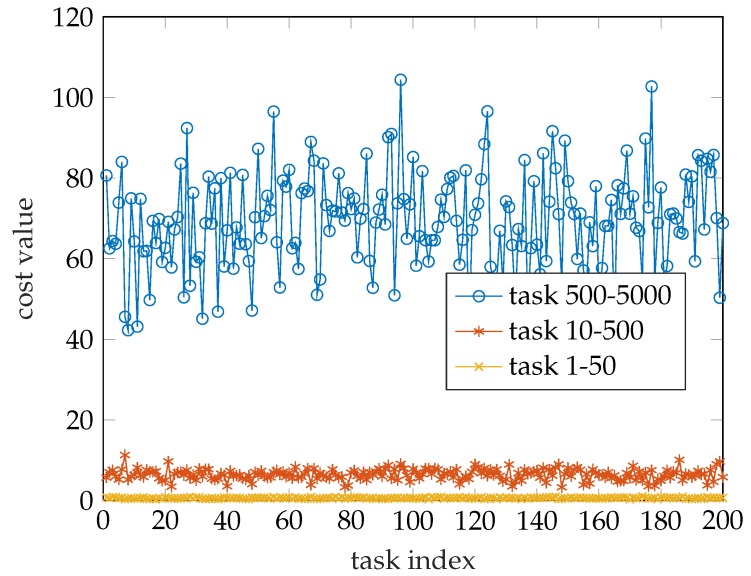
Cost value comparison.

**Figure 5:**
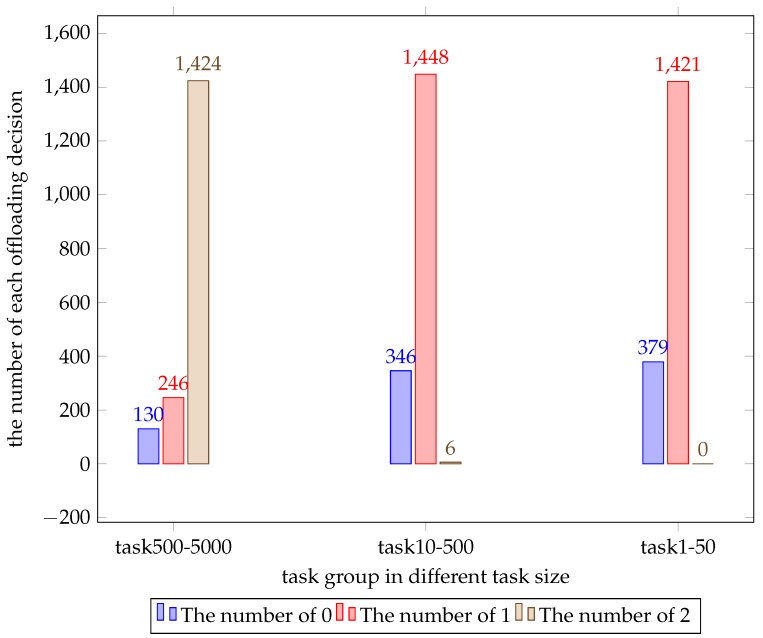
The number of each decision for each set of tasks.

Before studying the effects of the parameters, we think it is significant to study the change in the optimal offloading decision caused by the task size on different scales. We generated another two sets of random numbers that obey uniform distribution with 200 entries. One varies from 1 to 50, the other varies from 10 to 500. The cost value is shown in Figure 4. Their average cost values are 69.5, 6.4533 and 0.6245, respectively. In addition, we counted the number of each decision for each set of tasks and this is shown in Figure 5. It is obvious that the minimum cost value of the three sets of data fluctuates around the mean and the greater the variation range of the task size, the greater the fluctuation range. According to Figure 5, we can conclude that when the task size is generally large the WD tends to offload tasks to the cloud server to get a lower cost value.

We simulated different real environments by changing the parameters of the model. In this way, we could quantitatively study the variation of the optimal offloading decision under different environments. That is of great significance for industrial application, for example, we can dynamically adjust the number of edge servers or cloud servers according to currently optimal offloading decision when the environment changes. In this subsection, we consult industrial experts in this field and refer to relevant cases, finally we select the three most significant parameters α, β and the total upload bandwidth *W*. For simplicity, we denote the ratio of α to β as η. In the following experiment, we will assign specific values to η and *W*. For simplicity but without loss of generality, we randomly select 10 entries from above three task set as experimental data set. Table 2 lists the possible values of those parameters.

**Figure 6:**
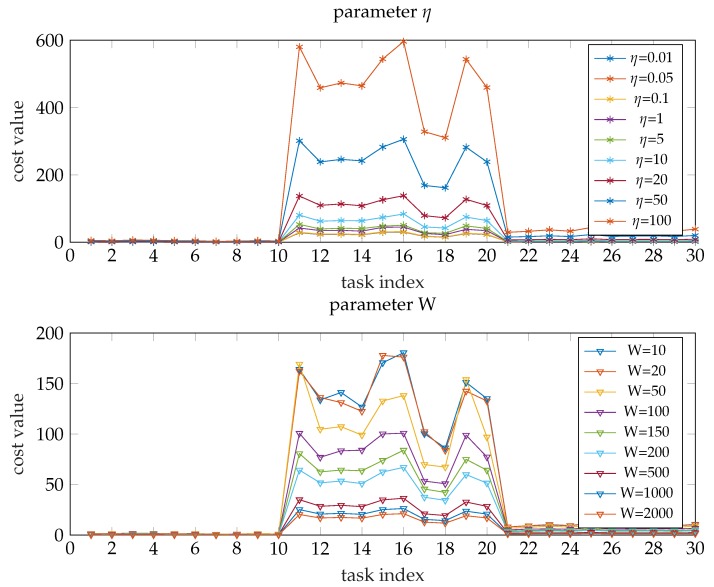
The cost values under different parameters.

Figure 6 shows the cost values under different parameters η and *W* (other parameters keep the initial value). It is obvious that the cost value is inversely proportional to *W* and directly proportional to η (this conclusion can also be drawn through analysing formula (18)).

**Figure 7:**
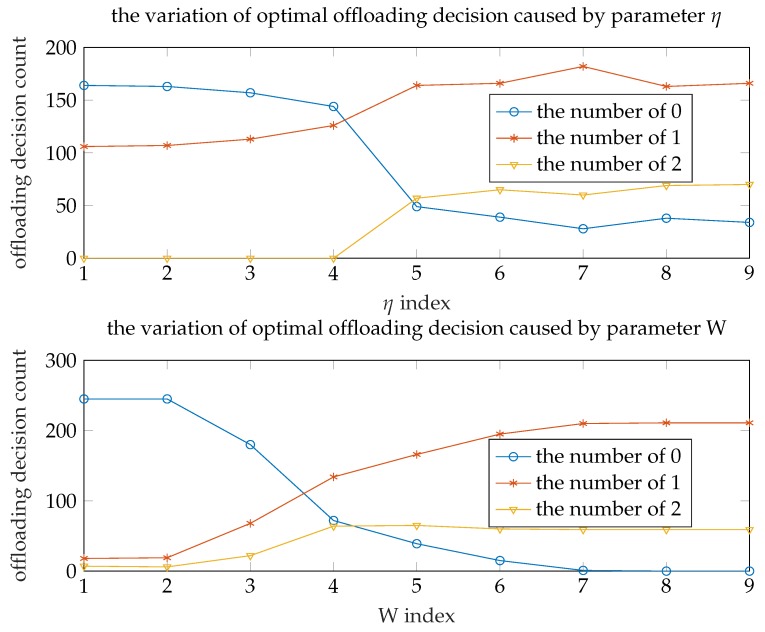
Offloading decision comparison.

Figure 7 shows the offloading decision count under different parameters η and *W* (other parameters keep the initial value). Each point in the figure represents the sum of WDs (*y* value) choosing a certain offloading decision (color) under current parameter(*x* value). It is obvious that there are no WDs choosing to offload their task to the edge server when the eta is relatively small, namely the energy consumption accounts for a small weight in the total cost. That is, we can reduce the number of cloud servers and add edge servers to improve system performance and save the financial expense. As the η increases, the number of three offloading decisions tends to steady and the number of WDs choosing to offload tasks to edge server is the largest. Based on this, we can deploy more edge servers to maximize system performance at energy-conscious scenario.

As the *W* decreases, the number of WDs choosing to execute locally will reduce to zero. That is, computing tasks are not performed locally. It means that if the bandwidth is big enough the terminal wireless device requires only very weak computing power to perform most tasks. 5G technology greatly improves the upload and download bandwidth. If the 5G technology and the mobile edge computing technology can be effectively combined, this can bring revolutionary change to the industry, for example, smart phones will become a display device with communication ability and weak computing power. This can greatly reduce the costs.

## 6. Conclusions

In this paper, we proposed a global cost model that takes time delay and energy consumption into account and formulated it into an optimization problem. Then, we employed two algorithms, enumeration and Branch-and-Bound, to resolve this optimization problem. While satisfying high accuracy, the Branch-and-Bound algorithm can get the result more quickly so it is more suitable for practical application. In addition, we simulated the changing trend of unloading decisions in different environments. In the future, we intend to combine artificial intelligence algorithms to further increase the speed of operation. 

## Figures and Tables

**Table 1:** Notations used in this paper.

N	the set of WDs
A	offloading action vector
$a_{n}$	offloading decision of the *n*-th WD
$f_{n}^{l}$	the computation capacity of the *n*-th WD (Mhz)
$f^{e}$	the computation capacity of edge server (Mhz)
$f^{c}$	the computation capacity of cloud server (Mhz)
$b_{n}^{w}$	the bandwidth between the *n*-WD and the edge server (Mbps)
*t*	total time delay introduced by core network
$B_{n}$	the size of the data to be transferred of the *n*-th WD (MB)
$D_{n}$	task size expressed as the total number of CPU cycles required to accomplish the computation task of the *n*-th WD.
θ	a constant $D_{n}=\theta B_{n}$
α	a constant $Q_{n}^{l}=\alpha E_{n}^{l}+\beta T_{n}^{l}$
β	a constant $Q_{n}^{l}=\alpha E_{n}^{l}+\beta T_{n}^{l}$
$P_{d}$	The full power of WD (W)
$P_{i}$	The idle power of WD (W)
$P_{t}$	The transmission power of WD (W)
$T_{n}^{l}$	the time delay of the *n*-th WD to executing computation task locally (s)
$T_{n}^{e}$	the time delay of the *n*-th WD to executing computation task on the edge server (s)
$T_{n}^{c}$	the time delay of the *n*-th WD to executing computation task on the cloud server (s)
$E_{n}^{l}$	the energy consumption of the *n*-th WD to executing computation task locally
$E_{n}^{e}$	the energy consumption of the *n*-th WD to executing computation on the edge server
$E_{n}^{c}$	the energy consumption of the *n*-th WD to executing computation task on the cloud server
$Q_{n}^{l}$	the total cost (the weighted sum of energy consumption and time delay ) of the *n*-th WD to executing computation task locally
$Q_{n}^{e}$	the total cost (the weighted sum of energy consumption and time delay ) of the *n*-th WD to executing computation task on the edge server
$Q_{n}^{c}$	the total cost (the weighted sum of energy consumption and time delay ) of the *n*-th WD to executing computation task on the cloud server
$Q_{all}$	the sum of $Q_{n}^{l},Q_{n}^{e}$ and $Q_{n}^{c}$
*W*	total upload bandwidth (MBps)

**Table 2:** The possible values of parameters.

η	[0.01, 0.05, 0.1, 1, 5, 10, 20, 50, 100]
*W*	[10, 20, 50, 100, 150, 200, 500, 1000, 2000]
